# Infectious Material of the Aphtha Epidemic

**Published:** 1897-10

**Authors:** 


INFECTIOUS MATERIAL OF THE APHTHA EPIDEMIC.—According to the *Deutsche Thierärztlche Wochenschrift* of October 12th of last year, Dr. Lydtin, Privy Councillor of Baden, had been informed by letter from J. St. Furtuna, chief of the veterinary department in Roumania, that Veterinary Inspector Starcovici, in the bacteriological institute of Dr. Babes, had succeeded, in February, 1896, in isolating a micro-organism from animals affected with foot-and-mouth disease, and that the same had been inoculated internally and subcutaneously into cattle, with the result that the foot-and-mouth disease was transmitted to them.—*Idem.*

